# Spatial Heterogeneity of *CYP9K1* Gene Overexpression Driving Cross‐Resistance to Insecticide in *Anopheles* Mosquitoes Across Sub‐Saharan Africa: A Systematic Review and Meta‐Analysis

**DOI:** 10.1155/japr/7708566

**Published:** 2026-05-30

**Authors:** Obinna Chukwuemeka Nwinyi, Kehinde Favour Siyanbola

**Affiliations:** ^1^ Biotechnology Research Cluster, Department of Biological Sciences, College of Science and Technology, Covenant University, Ota, Ogun State, Nigeria, covenantuniversity.edu.ng; ^2^ Covenant Applied Informatics and Communications African Centre of Excellence (CApIC-ACE), Ota, Ogun State, Nigeria

**Keywords:** *Anopheles*, cross-resistance, *CYP9K1*, cytochrome P450, insecticide resistance, malaria control, meta-analysis, molecular surveillance, pyrethroids, sub-Saharan Africa

## Abstract

Insecticide resistance in *Anopheles* mosquitoes poses a growing challenge to malaria elimination efforts across sub‐Saharan Africa, threatening the continued effectiveness of frontline interventions. Among the metabolic mechanisms driving resistance, the cytochrome P450 monooxygenase gene *CYP9K1* has been increasingly associated with detoxification and cross‐resistance to multiple insecticide classes, particularly pyrethroids. This review assessed spatial heterogeneity in *CYP9K1* overexpression (log₂ fold change) in cross‐resistant *Anopheles* mosquito populations across sub‐Saharan Africa. This systematic review was conducted in accordance with PRISMA guidelines, drawing data from PubMed, Scopus, Web of Science, ScienceDirect, BioMed Central, and Google Scholar (2015–2025). Random‐effects models using restricted maximum likelihood (REML) estimation were applied in JASP, alongside subgroup, sensitivity, and publication bias analyses. Out of 17,163 retrieved records, 11 studies met the inclusion criteria, representing data from six sub‐Saharan African countries. The pooled log_2_ fold change for *CYP9K1* expression was 1.910 (95% CI: 1.274–2.545; *p* < 0.001), confirming significant upregulation in resistant mosquito populations. Subgroup analyses further revealed that *CYP9K1* overexpression followed a similar trend across countries, with no statistically significant differences observed between the countries (*p* > 0.05). This consistency suggests that the same *CYP9K1*‐linked resistance mechanism may be spreading across different ecological and geographic regions, possibly through gene flow or shared selection pressure from insecticide use. These findings highlight *CYP9K1* as a key metabolic marker conferring cross‐resistance among *Anopheles* mosquitoes. The integration of *CYP9K1* molecular surveillance into national vector control programs will strengthen early detection of resistance hotspots, inform insecticide rotation policies, and support the development of next‐generation long‐lasting insecticidal nets (LLINs) incorporating synergists or nonpyrethroid active ingredients. This evidence‐based approach could guide tailored resistance management strategies essential for sustaining malaria control gains across sub‐Saharan Africa.

## 1. Introduction

Despite significant progress in control, malaria still remains a major public‐health threat in sub‐Saharan Africa as it still accounts for a high proportion of the world’s infectious disease morbidity and mortality [1, 2]. The World Health Organization [3] reported that there were increased cases of malaria in the world in the last few years, with the African Region being the worst hit, accounting for about 246 million cases out of 263 million globally. This case volume coupled with evidence of ongoing transmission of the disease indicates the necessity of vector control measures as a core of the malaria prevention strategies in the region. Although interventions based on insecticides, particularly long‐lasting insecticidal nets (LLINs) and indoor residual spraying (IRS), have resulted in a drastic decline in the incidence and mortality of malaria in regions where interventions have been adequately implemented, this progress is now threatened [4, 5].

Over the last few years, strategies for combating insecticide resistance have been implemented with the use of rotations and synergist combinations of insecticides (piperonyl butoxide, PBO) added on nets to prevent detoxification of these insecticides [6, 7]. Nonetheless, these solutions are persistently challenged by cross‐resistance: a phenomenon where the resistance to one insecticide causes or contributes to resistance to another. This diminishes the effect of rotating and synergist combinations of insecticide classes [8].

A major resistance mechanism is metabolic resistance, which involves the overexpression of detoxification enzymes, such as Cytochrome P450 monooxygenases (CYPs), glutathione‐S‐transferases (GSTs), and carboxylesterases (COEs), particularly in cases of cross‐resistance to multiple insecticide classes [9]. The P450s specifically have been studied in numerous functional and field studies due to their role in catalyzing oxidative reactions, which can detoxify a variety of insecticides [10, 11]. Given the broad substrate specificity of many P450 enzymes, elevated P450 activity can lead not only to resistance to pyrethroids but also cross‐resistance to organophosphates, carbamates, or other classes, especially when the same enzyme can metabolize more than one insecticide [12].

The most implicated insecticide resistance genes in the *Anopheles* species are the *CYP* genes. *CYP* genes such as *CYP6P3, CYP6M2, CYP6P9a/CYP6P9b, CYP6Z1, CYP6M7, CYP9K1* and others have been found to be overexpressed in specific species, regions and resistance contexts [13, 14]. Geographic distribution and dominance of specific *CYPs* are different: certain *CYPs* are found in dominance in southern Africa, others are found in West or East Africa, indicating histories of local selection pressure [15]. For example, *CYP6P9a* and *CYP6P9b* are highly overexpressed in *Anopheles funestus* populations in southern Africa (Malawi, Mozambique) and are associated with pyrethroid resistance [16]. Similarly, *CYP6M7* has been found upregulated in multiple locations, often alongside *CYP6P9* genes, indicating that multiple P450s may contribute together to resistance [17].

Among these CYP genes, *CYP9K1* has been identified as notable due to a growing body of evidence suggesting its role in cross‐resistance (in particular, pyrethroid) and its geographic distribution. Unlike many other CYPs that are primarily characterized by region‐specific overexpression, CYP9K1 is supported by evidence of a specific resistance‐associated mutation (G454A), providing a clearer mechanistic link to pyrethroid resistance. It is associated with resistance to multiple pyrethroids rather than a single compound, with stronger evidence linking it to Type II pyrethroids such as deltamethrin, while also showing involvement in resistance to Type I pyrethroids like permethrin, indicating its role in cross‐resistance across different pyrethroid classes. A recent investigation demonstrated that a single mutation (G454A) in the *CYP9K1* is closely linked with the pyrethroid resistance in *A. funestus*, and that the resistant allele has been distributed across Africa (Dominant in Uganda in 2014, followed by Cameroon in 2020) [18]. Also in Cameroon, studies have shown that *Anopheles* species resistant to both permethrin and deltamethrin show higher expression of *CYP9K1* among other genes, particularly in rainy seasons‐ suggesting that expression is not fixed but seasonally/ecologically variable [19]. Hearn et al. [20] also identified directional selection of a haplotype of *CYP9K1* in *A. funestus* conferring resistance to Type II pyrethroids (deltamethrin), as well as the moderate metabolism of DDT. The geographical pattern of CYP9K1 mutation frequency—that is, where *CYP9K1* mutation is fixed, where *CYP9K1* mutation is spreading, where *CYP9K1* mutation is rare‐implicates significant spatial heterogeneity in the applicability of *CYP9K1* mutation across countries in Africa [18]. Therefore, *CYP9K1* was selected for this study due to its well‐characterized resistance‐associated mutation (G454A), evidence of cross‐resistance across multiple pyrethroids, and its broader geographic distribution compared with other CYP genes.

Spatial heterogeneity in the expression levels of resistance genes, as regards the extent to which overexpression varies across countries, geographic areas, ecological space, and seasons, is critical to developing vector control strategies [21]. Furthermore, knowledge of spatial heterogeneity aids in anticipating the location of resistant alleles spreading, or whether that process is enhanced by gene flow, or the location of risk hotspots [22]. However, a significant gap persists, as although some studies indicate *CYP9K1* overexpression or mutation in particular populations, there has been no detailed, quantitative synthesis of the extent of *CYP9K1* gene overexpression (e.g. through log fold‐change values) in *Anopheles* populations across more than one country in sub‐Saharan Africa. The identification of spatial heterogeneity is crucial to ensure improved resistance surveillance, diagnostics, and the design of a strategy to control vectors.

Thus, this meta‐analysis curated published data on *CYP9K1* overexpression levels (log_2_ fold changes [FC]) in cross‐resistant *Anopheles* mosquito populations across sub‐Saharan Africa to assess spatial heterogeneity in expression levels and how they correlate with phenotypic cross‐resistance to different insecticide classes and their implications for molecular surveillance, intervention choice (e.g., choice of insecticide, use of synergists, nonpyrethroid alternatives), and policy in different geographic settings.

## 2. Methodology

To conduct this systematic review, published articles on insecticide resistance due to gene overexpression in *Anopheles* species were considered. The systematic review and meta‐analysis were conducted according to the Preferred Reporting Items of Systematic Reviews and meta‐analysis (PRISMA) guidelines [23]. The eligibility criteria were developed according to the relevance of the articles needed to achieve the objectives of this study.

### 2.1. Literature Search Strategy

A thorough systematic search of the literature was conducted across PubMed, Scopus, ScienceDirect, Biomed Central, Google Scholar, and Web of Science to identify articles related to gene expression and insecticide resistance in the *Anopheles* species. To identify more articles, the reference lists of eligible studies and reviews were also screened. To identify relevant studies, the search strategy was created by combining various keywords based on terms widely used in the literature on insecticide resistance, gene expression, and *Anopheles* mosquitoes. The search was performed using the following string for each database:

(“gene overexpression” OR “gene upregulation” OR “increased expression” OR “transcriptome” OR “transcriptomics” OR “RNA‐seq” OR “gene expression profiling” OR “qPCR” OR “microarray”)

AND (“insecticide resistance” OR “cross‐resistance” OR “multiple resistance” OR “metabolic resistance” OR “target‐site resistance” OR “resistance mechanisms” OR “detoxification enzyme”)

AND (“cytochrome P450” OR “CYP” OR “monooxygenase” OR “P450”)

AND (*Anopheles* OR “*Anopheles gambiae*” OR “*Anopheles funestus*” OR “*Anopheles arabiensis*” OR “*Anopheles stephensi*” OR “mosquito” OR “malaria vector”)

AND (“insecticide” OR “pyrethroid” OR “organophosphate” OR “carbamate” OR “DDT” OR “neonicotinoid”) AND (“Sub‐Saharan Africa” OR “Africa” OR “Nigeria” OR “Ghana” OR “Kenya” OR “Tanzania” OR “Uganda” OR “Ethiopia” OR “South Africa” OR “Burkina Faso” OR “Benin” OR “Cameroon” OR “Mozambique”).

### 2.2. Study Selection and Eligibility Criteria

All retrieved records were checked for duplicates before undergoing title, abstract, and full‐text screening in accordance with the defined inclusion and exclusion criteria.

Eligibility criteria were based on the PICO framework:

• Population: Natural or laboratory Anopheles populations (any life stage) with resistance phenotype defined by bioassay or field classification.
• Intervention/exposure: Insecticide exposure or defined resistant phenotype (survivors vs. dead; resistant strain vs. susceptible reference).
• Comparator: Susceptible mosquitoes (reference strain or susceptible field cohort).
• Outcomes: Gene expression metrics (log2 FC , fold change, means ± SD, raw counts) for individual genes.
• Study types: Experimental studies reporting gene expression by qPCR, microarray, RNA‐seq.

#### 2.2.1. Inclusion Criteria

The inclusion criteria of this study are as follows:

• Studies that compare gene expression, specifically *CYP9K1*, between resistant and susceptible *Anopheles* species of different insecticides in sub‐Saharan Africa.
• Studies that report or allow computation of effect size (log2FC or FC) and an uncertainty measure (SE, SD, CI, *p* value, *n*).
• Primary research articles, published in peer‐reviewed journals or preprints.
• Studies that use more than one insecticide.
• Studies published from 2015–2025
• Studies with full text available

#### 2.2.2. Exclusion Criteria

The exclusion criteria are described as follows:

• Studies that report gene expression levels in other mosquito species
• Studies that report gene expression levels in *Anopheles* species in countries not in sub‐Saharan Africa.
• Studies that report only proteomics without transcript‐level data.
• Studies that provide only qualitative claims without numerical gene‐expression data.
• Studies published before 2015.
• Studies not written in English.
• Studies with no access to full text.

Titles, abstracts, and full texts of retrieved records were independently screened by two reviewers, with any disagreements being resolved by a third reviewer consulted.

### 2.3. Data Extraction and Effect Size Calculation

A standardized data extraction form was used to extract the data from the studies such as StudyID, author, year, country, *Anopheles* species, insecticide, biological replicates (*n*), Gene symbol/ID, Effect size‐ Fold change (log_2_FC or FC), measure of uncertainty (SE, SD, CI, *p* value) (Table S1).

When *p* values were reported, SEs were derived using the formula:

(1) SE=Log2FCZ

where *Z*  was the standard normal deviate corresponding to the reported *p* value [24].

All effect sizes reported in FC were transformed to Log_2_FC for alignment across studies. In cases where studies reported more than one outcome, a single pooled effect size was calculated using inverse‐variance weighting such that each study provided only one independent estimate. The Log_2_FC of the pooled effect sizes was obtained as follows:

(2) ϴ∧pooled=∑wi×ϴ∧i ∑wi 

Where

Wi=1SEi

${\overset{\wedge }{\theta }}_{i}$ is the effect size (log_2_ FC) of each outcome in a study. *w*_*i*_ is the weight assigned to each outcome. SE*_i_* is the standard error of the effect size ${\overset{\wedge }{\theta }}_{i}$.

The pooled SE was calculated as follows:

(3) SEpooled=1∑wi

This was done according to Borenstein et al. [25] to prevent assigning more weight to studies with more outcomes than other studies to prevent an improper estimate of the precision of the summary effect.

### 2.4. Meta‐Analysis

Meta‐analysis was carried out using the JASP software (Version 0.95.1.0). A random‐effects meta‐analysis with the use of restricted maximum‐likelihood (REML) was conducted to check for heterogeneity because of the variability in populations and study designs. To determine between‐study heterogeneity, *τ* ^2^ (tau‐squared), which is the variance of true effects, and *I* ^2^, which is the proportion of variability explained by heterogeneity as opposed to sampling error, were calculated according to Cochrane recommendations [26]. To visualize the individual study effect sizes (Log_2_FC) with 95% confidence intervals and the pooled estimate, forest plots were generated to enable evaluation of study variation results.

#### 2.4.1. Subgroup Analysis

To achieve the main goal of the study, which is country‐level comparisons, a country subgroup analysis was conducted, with the differences between subgroups tested with a *χ* ^2^ test for subgroup heterogeneity [25].

#### 2.4.2. Publication Bias and Sensitivity Analyses

The potential effect of small studies and the effect of publication bias were evaluated with the funnel plots and the Egger regression test. The asymmetry of the funnel plot was analyzed based on the existing guidelines, and the trim‐and‐fill as well as the funnel plot analyses were interpreted to determine the possible effects of missing studies on pooled estimates [27]. Meta regression was conducted to validate publication bias results.

Sensitivity analyses were conducted to evaluate the robustness of the pooled effect size by comparing results obtained from fixed‐effect and random‐effects models. Changes in the pooled estimate across these scenarios were used to assess the stability of findings.

## 3. Results

### 3.1. Search Results

**Figure 1:**
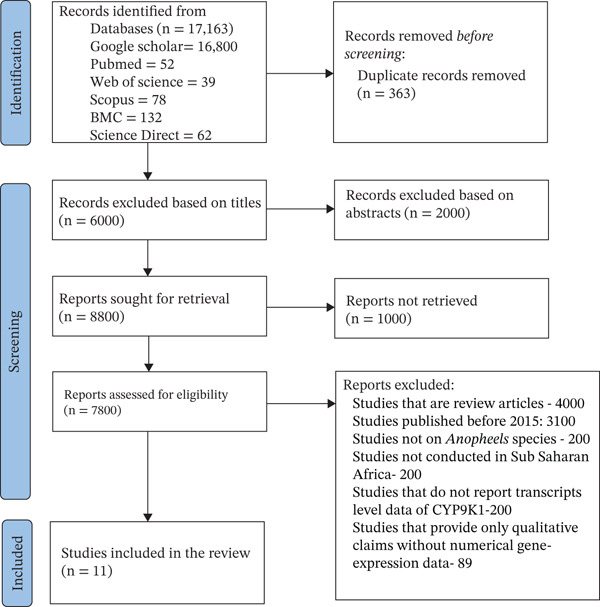
PRISMA flow diagram showing the selection process of eligible studies for this review.

A total of 17,163 studies were identified from several databases, including Google Scholar (16,800), PubMed (56), Web of Science (39), Scopus (78), BMC (132), and ScienceDirect (62), as shown in Figure 1. After which, 363 duplicates were removed, and 16,800 studies were screened using title and abstract. After title screening, 8000 of the studies were removed, and 2000 studies were removed after reviewing the abstracts, which means 7800 reports were subjected to full‐text evaluation. Among these, 4000 were filtered out as review articles, 3100 were filtered out because they were published prior to 2015. Another 600 studies were filtered out because they included studies that reported gene expression on other mosquito species, countries not in sub‐Saharan Africa, nor provided transcript‐level data of *CYP9K1*. Hundred eighty‐nine (189) were filtered out because they consist entirely of qualitative assertions with no numerical data of gene expression. Once all the eligibility criteria had been applied, 11 studies were included as part of the final review.

### 3.2. Study Characteristics

All the included studies reviewed show increasing molecular evidence of insecticide resistance in major malaria vectors in Africa, specifically *Anopheles* species (Table 1). The included studies geographically cover six countries in sub‐Saharan Africa, with most of the studies in West Africa, particularly in Nigeria, Ghana, Benin, and Cote d′Ivoire, which indicates a narrow scope of interest in the areas where malaria transmission is still very high. Central and East Africa were also represented, particularly in studies from Cameroon and Kenya. In these studies, pyrethroids (deltamethrin, alphacypermethrin, and permethrin) were the most tested insecticides, as they were used in almost all the studies, as they play a significant role in LLINs. Other insecticide classes, including organophosphates (e.g., malathion and pirimiphos‐methyl), carbamates (e.g., bendiocarb), and organophosphates (e.g., DDT) were also studied, indicating multi‐insecticide resistance among vectors.

**Table 1:** Study characteristics of eligible studies for this review.

Author	Country	Insecticide tested	Species	Assay type
Atoyebi et al. [28]	Nigeria	Permethrin, DDT	*Anopheles funestus*	Microarray, qRT‐PCR
Debrah et al. [29]	Kenya	Permethrin, deltamethrin	*A. funestus*	qRT‐PCR
Ibrahim et al. [30]	Nigeria, Niger, Chad and Cameroon.	Deltamethrin, permethrin	*Anopheles coluzzii*	qRT‐PCR
Kouadio et al. [31]	Côte d′Ivoire	Bendiocarb, deltamethrin, DDT and malathion	*Anopheles gambiae* sensu lato	qRT‐PCR
Miriam et al. [32]	Ghana	Permethrin, deltamethrin, Alphacypermethrin	*A. gambiae* sensu lato	qRT‐PCR
Mugenzi et al. [33]	Ghana	Deltamethrin, permethrin	*A. gambiae sensu lato, A. funestus*	qRT‐PCR
Omoke et al. [34]	Kenya	Deltamethrin (DELTA), alphacypermethrin (ACYP), and pirimiphos‐methyl (PMM)	*Anopheles arabiensis*	qRT‐PCR
Piameu et al. [35]	Cameroon	Deltamethrin, permethrin, alpha‐cypermethrin, and etofenprox	*A. gambiae sensu lato*	qRT‐PCR
Saizonou et al. [36]	Benin republic	Pirimiphos‐methyl (DP), alphacypermethrin (DA), or deltamethrin	*A. gambiae*	qRT‐PCR
Tchigossou et al. [37]	Benin republic	DDT, permethrin	*A. funestus*	Microarray, qRT‐PCR
Wipf et al. [38]	Côte d’Ivoire	Bendiocarb, deltamethrin, DDT, and malathion	*Anopheles coluzzii*	qRT‐PCR

The *Anopheles* mosquito species differed depending on the study, with *Anopheles gambiae* sensu lato and *A. funestus* being the most frequently studied. Such distribution is supported by their extensive geographic coverage and epidemiological significance in terms of malaria transmission. The most common assay method used throughout the studies was the quantitative real‐time polymerase chain reaction (qRT‐PCR), which was used to measure the expression of genes associated with metabolic or cuticular resistance. Some studies, such as Atoyebi et al. [28] and Tchigossou et al. (2018), complemented microarray analysis with qRT‐PCR to give more comprehensive transcriptomic data. All these studies revealed consistent trends in overexpression of cytochrome P450S, GSTs and cuticular proteins as mediators of metabolic resistance across countries in sub‐Saharan Africa.

### 3.3. Meta‐Analysis Results

#### 3.3.1. Pooled Effect Sizes

The overall pooled log FC estimate (Table 2) was calculated from the multiple outcome results presented in Supplementary materials, which summarized log fold change values and standard errors obtained from all studies included.

**Table 2:** Overall pooled log fold change estimates for included studies.

Study ID	Country	Pooled LogFC	Pooled SE	*p* value
Atoyebi et al. [28]	Nigeria	1.388	0.659	*p* ≤ 0.05
Debrah et al. [29]	Kenya	3.445	1.762	*p* < 0.05
Omoke et al. [34]	Kenya	1.567	0.370	*p* < 0.05
Ibrahim et al. [30]	Nigeria	1.571	0.802	*p* < 0.05
Ibrahim et al. [30]	Cameroon	1.758	0.897	*p* < 0.05
Kouadio et al. [31]	Côte d′Ivoire	2.32	0.064	*p* < 0.001
Wipf et al. [38]	Côte d′Ivoire	3.495	0.863	*p* < 0.001
Miriam et al. [32]	Ghana	3.344	0. 98	*p* < 0.05
Mugenzi et al. [33]	Ghana	0.242	0.108	*p* < 0.05
Saizonou et al. [36]	Benin Republic	2.47	0.332	*p* < 0.01
Tchigossou et al. [37]	Benin Republic	1.123	0.474	*p* ≤ 0.05
Piameu et al. [35]	Cameroon	2.76	0.966	*p* ≤ 0.05

#### 3.3.2. Heterogeneity Statistics: *τ* ^2^, *I* ^2^, and Q‐Test *p* Value

The random‐effects meta‐analysis yielded a pooled Log_2_FC estimate of 1.910 (95% CI: 1.274‐2.545; *p* < 0.001), which is statistically significant when comparing overexpression of the target gene *CYP9K1* in the resistant mosquito populations to the susceptible ones. The heterogeneity test was significant (*Q*_*e*_(11) = 290.57; *p* < 0.001), indicating a lot of variation in effect sizes across studies, as depicted in Table 3. In addition, the between‐study variance was significant (*τ* = 0.854; *τ* ^2^ = 0.729) and the proportion of true heterogeneity was high (*I* ^2^ = 92.0*%*; 95% CI: 77.6%–97.5%) as shown in Table 4, indicating that most of the observed variation was due to real differences between studies as the results may vary in different settings and populations and not sampling error. The 95% prediction interval (–0.074 to 3.893) indicates that, although the average effect is significant, individual future studies may show smaller or even null effects.

**Table 3:** Tests of heterogeneity and overall pooled effect.

Test	Value	*p* value
Heterogeneity	*Q*_*e*_(11) = 290.57	< 0.001
Pooled effect	*t*(11) = 6.62	< 0.001

**Table 4:** Meta‐analytic estimates of effect size and variability.

	Estimate	95% CI (lower–upper)	95% prediction interval (lower–upper)
Pooled effect	1.910	1.274–2.545	–0.074–3.893
Between‐study SD (*τ*)	0.854	0.469–1.557	
Between‐study variance (*τ* ^2^)	0.729	0.220–2.424	
Heterogeneity (*I* ^2^ %)	92.01	77.63–97.45	

#### 3.3.3. Forest Plot

**Figure 2:**
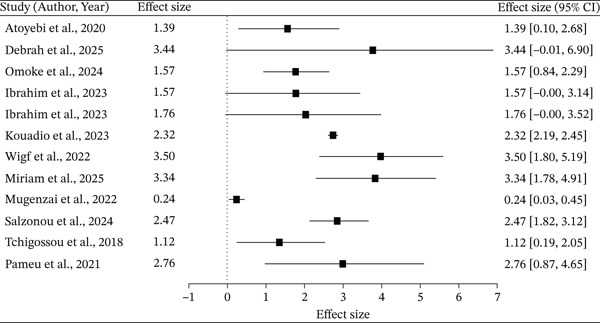
Forest plot showing individual study effects and the pooled estimate based on the random effects model.

The forest plot (Figure 2) shows individual study effect sizes, represented by squares proportional to study weight, with 95% confidence intervals, were mostly positive. The effect sizes in individual studies range from small (e.g., 0.24 [0.03, 0.45]) to large (e.g., 3.44 [−0.01, 6.90]). A number of studies reported statistically significant positive effects (e.g., 2.32 [2.19, 2.45]), 0.24 [0.03, 0.45]), whereas some studies were not significant, as their confidence intervals crossed zero (e.g., 1.57 [−0.00, 3.14]; 3.44 [−0.01, 6.90]). The combined outcomes of the studies indicate a positive trend across the effect sizes, but the heterogeneity across studies is moderate to high.

#### 3.3.4. Subgroup Analyses

Subgroup meta‐analysis indicated that there were no statistically significant country differences (*Q*_*m*_(5) = 7.44, *p* = 0.190) as shown in Table 5, meaning that country did not have any significant effect on the degree of gene over‐expression with insecticide resistance. However, within‐country heterogeneity was low in Nigeria (*Q*_*e*_ = 0.03, *p* = 0.860), Kenya (*Q*_*e*_ = 1.09, *p* = 0.297), Cameroon (*Q*_*e*_ = 0.58, *p* = 0.447), and Côte d′Ivoire (*Q*_*e*_ = 1.84, *p* = 0.175) showing no significant heterogeneity, whereas Ghana (*Q*_*e*_ = 14.84, *p* < 0.001) and Benin Republic (*Q*_*e*_ = 5.42, *p* = 0.020) exhibited substantial variability among studies. The pooled effect was statistically significant only in Nigeria (*t* = 16.28, *p* = 0.039), with an estimate of 1.462 (95% CI: 0.321–2.602), indicating a clear positive effect, whereas pooled effects in Kenya, Cameroon, Côte d′Ivoire, Ghana, and Benin were not significant, and their confidence intervals were wide, often including zero as shown in Table 6. These values do imply that although the effect sizes were mostly similar among countries, within‐country variation could be due to local environmental or genetic effects on gene expression.

**Table 5:** Subgroup meta‐analysis by country showing heterogeneity and pooled effects of gene overexpression on insecticide resistance.

Analysis type	Country	Test statistic	*p* value
Heterogeneity	Nigeria	*Q*_*e*_(1) = 0.03	0.860
	Kenya	*Q*_*e*_(1) = 1.09	0.297
	Cameroon	*Q*_*e*_(1) = 0.58	0.447
	Côte d′Ivoire	*Q*_*e*_(1) = 1.84	0.175
	Ghana	*Q*_*e*_(1) = 14.84	< 0.001
	Benin Republic	*Q*_*e*_(1) = 5.42	0.020

Pooled effect	Nigeria	*t*(1) = 16.28	0.039
	Kenya	*t*(1) = 3.38	0.183
	Cameroon	*t*(1) = 4.45	0.141
	Côte d′Ivoire	*t*(1) = 5.23	0.120
	Ghana	*t*(1) = 1.09	0.472
	Benin Republic	*t*(1) = 2.74	0.223

Subgroup differences		*Q*_*m*_(5) = 7.44	0.190

**Table 6:** Meta‐analytic estimates of pooled effect sizes, confidence and prediction intervals, and heterogeneity by country.

Analysis	Subgroup	Estimate	95% CI lower	95% CI upper	95% PI lower	95% PI upper
Pooled effect	Nigeria	1.462	0.321	2.602	0.321	2.602
	Kenya	1.716	–4.731	8.162	–6.321	9.753
	Cameroon	2.222	–4.126	8.570	–4.126	8.570
	Côte d′Ivoire	2.592	–3.707	8.892	–6.931	12.115
	Ghana	1.692	–17.973	21.358	–31.642	35.026
	Benin Republic	1.839	–6.702	10.380	–12.031	15.709

*τ*	Nigeria	0.000	0.000	4.063		
	Kenya	0.378	0.000	10.000		
	Cameroon	0.000	0.000	10.000		
	Côte d’Ivoire	0.562	0.000	10.000		
	Ghana	2.118	0.796	10.000		
	Benin Republic	0.860	0.115	10.000		

*τ* ^2^	Nigeria	0.000	0.000	16.511		
	Kenya	0.143	0.000	100.000		
	Cameroon	0.000	0.000	100.000		
	Côte d′Ivoire	0.316	0.000	100.000		
	Ghana	4.487	0.633	100.000		
	Benin Republic	0.740	0.013	100.000		

*I* ^2^ (%)	Nigeria	0.000	0.000	96.840		
	Kenya	8.090	0.000	98.405		
	Cameroon	0.000	0.000	99.139		
	Côte d′Ivoire	45.759	0.000	99.627		
	Ghana	93.261	66.144	99.677		
	Benin Republic	81.542	7.270	99.833		

**Figure 3:**
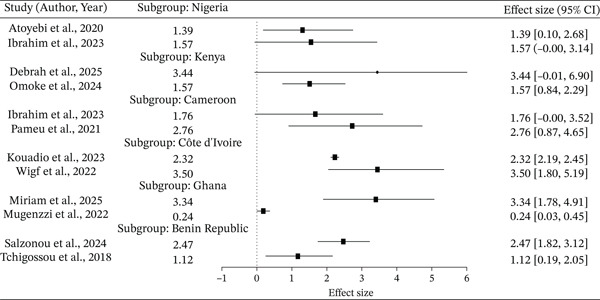
Forest plot showing the pooled estimate by country, based on the random‐effects model.

The forest plot (Figure 3) shows generally positive associations between gene overexpression and insecticide resistance across countries, with variation in strength and significance. One study found a significant effect (*E* *S* = 1.39, 95% CI: [0.10–2.68]) and one did not (ES = 1.57, 95% CI: [−0.00–3.14]) in Nigeria, and significant effects (ES = 3.44, 95% CI: [−0.01–6.90]) and nonsignificant effects (ES = 1.57, 95% CI: [0.84–2.29]) in Kenya. Significant positive effects were always observed in Cameroon (ES = 1.76 [−0.00–3.52]; 2.76 [0.87–4.65]) and Cote d′Ivoire (ES = 2.32 [2.19–2.45]; 3.50 [1.80–5.19]) and the Benin Republic (ES = 2.47 [1.82–3.12]; 1.12 [0.19–2.05]). Mixed results were obtained in Ghana, where one study was significant (ES = 3.34 [1.78–4.91]) and another was not (ES = 0.24 [0.03–0.45]).

This analysis demonstrates the significant overexpression of the gene in *Anopheles* mosquitoes across all Saharan Africa regions, with the strength of association being consistent across countries despite some within‐country variability.

#### 3.3.5. Publication Bias

**Figure 4:**
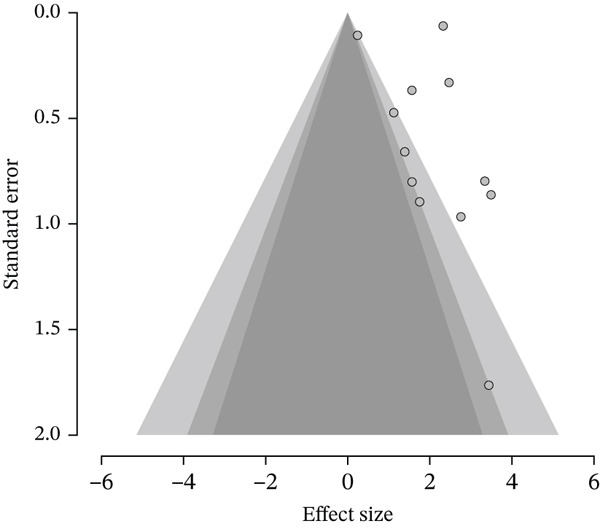
Funnel plot of included studies assessing CYP9K1 overexpression, with visual assessment of publication bias.

To determine possible publication bias in included studies, the funnel plot (Figure 4) was generated. A visual examination showed that there was an apparent asymmetry, with the majority of the studies located to the right‐hand side, which implies that the majority of the effects were positive, whereas there were only a small number of studies found on the left. A small study, which is at the bottom of the plot, was also subject to small study effects by reporting larger effect sizes. Further statistical tests were carried out to further assess the bias, and the meta regression test for funnel plot asymmetry (Table 7) showed no significant asymmetry (*z* = 1.622, *p* = 0.105, *μ* = 1.228, 95% CI: 0.236–2.219). Similarly, Egger′s weighted regression test (Table 8) showed no statistically significant asymmetry (*t* = –0.098, df = 10, *p* = 0.924, *μ* = 1.821, 95% CI: 1.016–2.625), indicating that small‐study effects or selective reporting may have limited influence on the pooled estimate.

**Table 7:** Meta‐regression test for funnel plot asymmetry.

	Asymmetry test		Limit estimate *μ*		
Estimates	*z*	*p*	Limit estimate *μ*	95% CI lower	95% CI upper
12	1.622	0.105	1.228	0.236	2.219

**Table 8:** Egger′s weighted regression test for funnel plot asymmetry.

	Asymmetry test			Limit estimate *μ*		
Estimates	t	df	p	Estimate	95% CI lower	95% CI upper
12	–0.098	10	0.924	1.821	1.016	2.625

#### 3.3.6. Sensitivity Analyses

A sensitivity analysis was performed to determine the robustness of the pooled estimates under fixed‐effects and random‐effects. A similar estimate of 1.799 (95% CI: 1.203–2.395; *p* < 0.001) was obtained using the pooled Log_2_FC estimate of the fixed‐effect model (Tables 9 and 10) to the pooled Log_2_FC estimate of the random‐effects model 1.910 (95% CI: 1.274–2.545; *p* < 0.001) (Tables 3 and 4). The similarity in statistical significance of these estimates suggests that the overall result of the study findings is not affected by the model used.

**Table 9:** Meta‐analytic tests for heterogeneity and pooled effect using the fixed effects model.

Test	Statistic	*p* value
Heterogeneity	*Q*_*e*_(11) = 290.57	< 0.001
Pooled effect	*t*(11) = 6.64	< 0.001

**Table 10:** Meta‐analytic pooled effect estimates using fixed effects model.

	95% CI			95% PI	
Estimate	Lower	Upper	Lower	Upper	Lower
Pooled effect	1.799	1.203	2.395		

## 4. Discussion

It has been documented in studies that the degree of gene overexpression associated with insecticide resistance in *Anopheles* mosquitoes can be greatly influenced by geographic or country‐level factors [39–41]. This is based on the understanding that various malaria endemic areas in Africa have diverse ecological settings and approaches to vector control, which may result in a heterogeneous pattern of resistance. An example is that intensive users of pyrethroid‐treated LLINs or IRS may cause stronger selection pressure on resistance genes than those with lower utilization, potentially resulting in differential patterns of gene expression [42]. The data obtained from the result of this indicated the consistent overexpression of *CYP9K1* in conferring cross‐resistance to insecticides in *Anopheles* mosquitoes across six sub‐Saharan African countries, irrespective of differences in local contexts. However, subgroup analyses showed that the between‐country difference test was not statistically significant (*Q*_*m*_(5) = 7.44, *p* = 0.190) (Table 3), indicating that the level of *CYP9K1* gene overexpression on resistance is widely similar among the six African countries studied, namely Nigeria, Ghana, Benin Republic, Kenya, Cameroon and Cote d′Ivoire.

The consistent overexpression of *CYP9K1* across all countries studied corresponds to the already documented association of the gene with cross‐resistance to insecticides such as deltamethrin and permethrin, the primary active compounds in LLINs and IRS. Studies have demonstrated that *CYP9K1* is very effective in metabolizing pyrethroids, conferring both pyrethroid resistance and cross‐resistance, and is often overexpressed in the resistant mosquitoes of *A. gambiae* and *Anopheles coluzzii* [30, 33]. The overexpression of *CYP9K1* correlates with less susceptibility to deltamethrin as well as permethrin, and other insecticides like DDT and some carbamates [28, 32]. The occurrence of such patterns further serves as evidence that extensive usage of pyrethroids has served as a strong selection pressure both in terms of fostering resistance to pyrethroids and in predisposing mosquito populations to cross‐resistance, which can weaken future control efforts. The *CYP9K1* overexpression demonstrated increased resistance in *Anopheles* species both to deltamethrin and permethrin, as well as partial resistance to pyriproxyfen, a juvenile hormone analogue that is being more regularly incorporated in next‐generation LLINs [28]. This indicates that overexpression of *CYP9K1* could also result in resistance to multiple insecticides, thereby diminishing the effect of these interventions. In addition, *CYP9K1* has been reported to be coexpressed with numerous other detoxification genes, including *CYP6P3* and *GSTe2*, in several resistant populations, further conferring cross‐resistance in *Anopheles* species [33, 43].

The observed consistency in *CYP9K1* overexpression across different ecological and geographical contexts also suggests that resistance is not purely local but shaped by regional factors, including the widespread use of similar intervention tools. This indicates that the extensive use of insecticide‐based interventions across sub‐Saharan African countries could have led to a uniform selection pressure, which favors the independent evolution or retention of this metabolic resistance phenotype across different *Anopheles* mosquito populations [5, 44]. Even though some countries, such as Ghana and the Benin Republic, exhibited high within‐country variability in gene expression (*I* ^2^ > 80*%*), this did not translate into systematic differences between countries. This shows that although local environmental or operational factors might generate variability within regions, these influences are not strong enough to produce divergent resistance patterns at the national scale. It also aligns with population genomics data showing there is a large degree of gene flow between the *Anopheles* populations in Africa. Several studies have shown that populations of *A. gambiae* are both highly migratory and low in genetic differentiation, enabling resistance alleles to rapidly spread to large geographical distances without being trapped by the effects of isolation‐by‐distance [45, 46]. Tagne et al. [18] reported on the predominance of the same haplotype of *454A-CYP9K1* resistant allele, which has likely spread from East to West across the Equatorial zone of Africa. Thus suggesting extensive gene flow between populations from Uganda to Cameroon, contributing to the overexpression of the gene in African countries. The resistance observed is not simply the result of independent, local adaptations but rather the product of shared selective pressures and genetic connectivity across regions.

However, not all studies describe consistent patterns of *CYP9K1* expression. Some have reported high levels of geographic heterogeneity. As an example, some studies in parts of East Africa have recorded inconsistent levels of *CYP9K1* upregulation, where certain communities have reported high levels of the up‐regulation in other communities, whereas the West Africa have exhibited very low levels despite high pyrethroid pressure [18, 47, 48]. This inconsistency can be due to the interplay of more than one type of resistance, for example, target‐site mutations like *kdr* (knockdown resistance) mutations, behavioral avoidance mechanisms, or another type of detoxification, for example, GSTs and esterases [20]. Moreover, environmental conditions, the use of the local mosquito insecticides and variations in the composition of mosquito species may also moderate the proportionate contribution of *CYP9K1* in the overall phenotypes of resistance. For instance, Riveron et al. [49] have reported the difference in the levels of metabolic resistance gene expression among *A. funestus* populations in various areas of southern and eastern Africa. This type of variability is significant as it demonstrates that although *CYP9K1* is a key factor in most situations, resistance is a complex phenomenon. There is a possibility of cross‐resistance not only by *CYP9K1* alone but a combination of several metabolic genes and their interaction with target‐site mutations [50]. There are also species differences that contribute to the formation of heterogeneous resistance profiles. Although *CYP9K1* is a significant contributor to resistance in *A. gambiae* and *A. coluzzii*, resistance in *A. funestus* is commonly linked to other P450 gene families, especially *CYP6P9a* and *CYP6P9b* as key metabolic resistance genes in this insect [51]. Such species‐specific variations imply that the evolution of resistance does not progress the same way in all vectors and that the most predominant mechanisms can differ based on the species composition in a specific region. This has far‐reaching consequences on the control programs that need to strategize programs depending on the species available in various geographical locations.

The consistency observed in CYP9K1 overexpression across Nigeria, Kenya, Cameroon, Cote d′Ivoire, Ghana, and the Benin Republic indicates a specific combination of factors in the sampled regions capturing broader continental patterns rather than local variations. Intense campaigns of LLINs and IRS with similar insecticide formulations have been conducted over a long period in all six countries studied (Nigeria, Kenya, Cameroon, Cote d′Ivoire, Ghana, and the Benin Republic) with similar selective environments [40]. Conversely, there has been extensive human movement and trade between these countries, which promotes the spread of mosquitoes and exchange of genes (gene flow). The combination of uniform selection pressures and high connectivity most likely homogenized resistance profiles, resulting in the consistent *CYP9K1* overexpression observed. Studies that reported heterogeneity were performed at finer geographic scales, within one country, or one region when local environmental factors have a more significant impact. In comparison, the current study deals with cross‐country trends, in which continent‐wide selection pressures caused by homogenous vector control interventions are probably the most significant signal. In addition, high gene flow among mosquitoes′ populations likely smooths out local differences over time, leading to continent‐wide homogenization for highly advantageous alleles like those associated with *CYP9K1*. Consequently, *CYP9K1* overexpression has emerged as a common aspect of most *Anopheles* populations even if other resistance mechanisms differ locally. However, it is important to acknowledge that this study focused on a single gene, and insecticide resistance is a complex, polygenic trait involving multiple mechanisms. Target‐site mutations such as L1014F kdr, other P450 enzymes, esterases, and GSTs all contribute to resistance, and their expression patterns may vary geographically even if *CYP9K1* appears uniform. Additionally, this study sampled specific locations, and the findings may not be generalizable to regions with different ecological or operational contexts. Integrating whole‐genome analyses, ecological data, and longitudinal monitoring could provide a broader picture of how resistance evolves and spreads.

## 5. Implications and Future Directions

The results from this review could provide significant implications for the design and implementation of insecticide resistance surveillance programs in Africa. It is obvious that *CYP9K1* has been overexpressed consistently across different Africa countries; this underscores importance of molecular surveillance systems that can monitor the key resistance genes in real time. Traditional phenotypic bioassays (e.g., WHO tube tests or CDC bottle assays) continue to play roles in identifying the changes in the susceptibility status; however, they may be inadequate to elucidate the underlying metabolic pathways of resistance. The integration of the molecular markers such as *CYP9K1*, *CYP6P3*, *GSTe2*, and *kdr* mutations into the national surveillance systems would offer advance‐warning systems of the developing trends of resistance before they can be reflected in the failure of field control [52].

It can be deduced that the uniform overexpression of CYP9K1 across diverse ecological zones suggests that resistance alleles are spreading rapidly due to gene flow and sustained selection pressure. This becomes problematic to the traditional approach of focusing surveillance efforts on localized sentinel sites. It is certain that resistance monitoring must adopt a regional or cross‐border perspective, recognizing that resistance dynamics often transcend national boundaries. Strengthening regional networks, such as those coordinated through WHO and PMI VectorLink, can help ensure that data are shared across countries, enabling timely responses to emerging resistance hotspots. In addition, the presence of high within‐country heterogeneity underscores the importance of maintaining fine‐scale surveillance alongside broader monitoring efforts.

Although country‐level differences may not be statistically significant, local ecological or operational factors such as variations in vector species composition, agricultural insecticide use, or implementation quality of control programs can still shape resistance dynamics in meaningful ways. Therefore, surveillance systems should combine continent‐wide molecular markers like *CYP9K1* with localized entomological and operational data to provide a comprehensive understanding of resistance evolution.

## 6. Conclusion

The data obtained in study has shown strong evidence for continent‐wide selective forces driving uniform *CYP9K1* overexpression in *Anopheles* mosquitoes, thus reflecting the pervasive impact of pyrethroid‐based interventions. This has significant implications for malaria control, as cross‐resistance can severely limit the effectiveness of alternative insecticides intended for resistance management. If *CYP9K1* and similar genes continue to spread and diversify, they could compromise not only current pyrethroid‐based strategies but also different insecticidal chemical classes. Monitoring *CYP9K1* expression alongside other metabolic and target‐site markers will be crucial for predicting resistance trends and designing interventions that can outpace the adaptive potential of mosquito populations.

## Author Contributions

Obinna Chukwuemeka Nwinyi: conceptualization, data curation, supervision, review and editing. Kehinde Favour Siyanbola: writing—original draft, data curation, data analysis.

## Funding

No funding was received for this manuscript.

## Conflicts of Interest

The authors declare no conflicts of interest.

## Supporting information


**Supporting Information 1** Additional supporting information can be found online in the Supporting Information section. The supporting data provide a detailed summary of all studies included in the meta‐analysis assessing gene overexpression associated with cross‐insecticide resistance in *Anopheles* mosquitoes across Africa. The table presents extracted study characteristics, including author and publication year, country of study, biological replicate number, pool size and pooling method, reported gene expression values (Log_2_ fold change), statistical significance (*p* values), and corresponding measures of uncertainty (standard error). These data were used to calculate the effect sizes and confidence intervals presented in the forest plot, ensuring transparency and reproducibility of the meta‐analysis.

## Data Availability

The data that support the findings of this study are available from the corresponding authors upon reasonable request.
