# Supplementary material for: Spatial Heterogeneity of CYP9K1 Gene Overexpression Driving Cross‐Resistance to Insecticide in Anopheles Mosquitoes Across Sub‐Saharan Africa: A Systematic Review and Meta‐Analysis
Source: J Parasitol Res. 2026 May 30;2026:7708566. doi: 10.1155/japr/7708566 (PMC13238507; doi:10.1155/japr/7708566)
Supplement: Supplementary file 1 — Supporting Information 1 Additional supporting information can be found online in the Supporting Information section. The supporting data provide a detailed summary of all studies included in the meta‐analysis assessing gene overexpression associated with cross‐insecticide resistance in Anopheles mosquitoes across Africa. The table presents extracted study characteristics, including author and publication year, country of study, biological replicate number, pool size and pooling method, reported gene expression values (Log2 fold change), statistical significance (p values), and corresponding measures of uncertainty (standard error). These data were used to calculate the effect sizes and confidence intervals presented in the forest plot, ensuring transparency and reproducibility of the meta‐analysis. [file JAPR-2026-7708566-s001.docx]

**Supplementary information**

**Table 1:** Summary of included studies data extracted including Author, Year, Country, *Anopheles* species, Insecticide, biological replicates(n), Gene symbol/ID, Effect size- Fold change (log_2_FC or FC), Measure of uncertainty (SE, SD, CI, p-value)

| **Study ID** | **Country** | **Biological replicates (n)** | **Pool size** | **Pooled type** | **Log_2_ FC** | **P value** | **SE** |
| --- | --- | --- | --- | --- | --- | --- | --- |
| Atoyebi et al., 2020 | Nigeria | 3 | 10 | Pool | 3.18 | P≤0.05 | 1.627 |
| Atoyebi et al., 2020 | Nigeria | 3 | 10 | Pool | 1.41 | P≤0.05 | 0.721 |
| Debrah et al., 2025 | Kenya | 8 | 10 | Pool | 3.445 | P<0.05 | 1.762 |
| Omoke et al., 2024 | Kenya | 3 | 10 | Pool | 1.3785 | P<0.05 | 0.705 |
| Omoke et al., 2024 | Kenya | 3 | 10 | Pool | 1.8197 | P<0.05 | 0.929 |
| Omoke et al., 2024 | Kenya | 3 | 10 | Pool | 1.872 | P<0.05 | 0.955 |
| Omoke et al., 2024 | Kenya | 3 | 10 | Pool | 1.5607 | P<0.05 | 0.796 |
| Omoke et al., 2024 | Kenya | 3 | 10 | Pool | 1.6088 | P<0.05 | 0.820 |
| Ibrahim et al., 2023 | Nigeria | 3 | 8 | Pool | 1.571 | P<0.05 | 0.802 |
| Ibrahim et al., 2023 | Cameroon | 3 | 8 | Pool | 1.758201 | P<0.05 | 0.897 |
| Kouadio et al., 2023 | Côte d’Ivoire | 50 | N/A | Individual | 5.3 | P<0.001 | 0.301 |
| Kouadio et al., 2023 | Côte d’Ivoire | 50 | N/A | Individual | 4.3 | P<0.001 | 0.244 |
| Kouadio et al., 2023 | Côte d’Ivoire | 50 | N/A | Individual | 2.8 | P<0.001 | 0.159 |
| Kouadio et al., 2023 | Côte d’Ivoire | 50 | N/A | Individual | 5.2 | P<0.001 | 0.295 |
| Kouadio et al., 2023 | Côte d’Ivoire | 50 | N/A | Individual | 2.0 | P<0.001 | 0.114 |
| Kouadio et al., 2023 | Côte d’Ivoire | 50 | N/A | Individual | 1.9 | P<0.001 | 0.108 |
| Wipf et al, 2022 | Côte d’Ivoire | 5 | N/A | pooled | 3.6 | P<0.001 | 1.734 |
| Wipf et al., 2022 | Côte d’Ivoire | 5 | N/A | pooled | 3.4 | P<0.001 | 1.638 |
| Wipf et al, 2022 | Côte d’Ivoire | 5 | N/A | pooled | 2.6 | P<0.001 | 1.252 |
| Miriam et al., 2025 | Ghana | 3 | N/A | Pooled | 3.342 | P<0.05 | 1.705 |
| Miriam et al., 2025 | Ghana | 3 | N/A | Pooled | 5.633 | P<0.05 | 2.875 |
| Miriam et al., 2025 | Ghana | 3 | N/A | Pooled | 3.475 | P<0.05 | 1.774 |
| Miriam et al., 2025 | Ghana | 3 | N/A | Pooled | 4.7965 | P<0.05 | 2.450 |
| Miriam et al., 2025 | Ghana | 3 | N/A | Pooled | 7.48 | P<0.05 | 3.820 |
| Miriam et al., 2025 | Ghana | 3 | N/A | Pooled | 5.983 | P<0.05 | 3.056 |
| Miriam et al., 2025 | Ghana | 3 | N/A | Pooled | 4.442 | P<0.05 | 2.268 |
| Miriam et al., 2025 | Ghana | 3 | N/A | Pooled | 3.969 | P<0.05 | 2.025 |
| Mugenzi et al., 2022 | Ghana | 3 | 10 | Pooled | 0.27 | P<0.05 | 0.138 |
| Mugenzi et al., 2022 | Ghana | 3 | 10 | Pooled | -0.6439 | P<0.05 | 0.328 |
| Mugenzi et al., 2022 | Ghana | 3 | 10 | Pooled | 0.4114 | P<0.05 | 0.210 |
| Mugenzi et al., 2022 | Ghana | 3 | 10 | Pooled | 3.3505 | P<0.05 | 1.671 |
| Mugenzi et al., 2022 | Ghana | 3 | 10 | Pooled | 3.654 | P<0.05 | 1.823 |
| Mugenzi et al., 2022 | Ghana | 3 | 10 | Pooled | 2.651 | P<0.05 | 1.323 |
| Saizonou et al., 2024 | Benin Republic | 3 | 7 | Pooled | 1.228 | P<0.01 | 0.477 |
| Saizonou et al., 2024 | Benin Republic | 3 | 7 | Pooled | 1.944 | P<0.01 | 0.754 |
| Saizonou et al., 2024 | Benin Republic | 3 | 7 | Pooled | 2.648 | P<0.01 | 1.026 |
| Saizonou et al., 2024 | Benin Republic | 3 | 7 | Pooled | 2.600 | P<0.01 | 1.007 |
| Saizonou et al., 2024 | Benin Republic | 3 | 7 | Pooled | 3.375 | P<0.01 | 1.307 |
| Saizonou et al., 2024 | Benin Republic | 3 | 7 | Pooled | 2.851 | P<0.01 | 1.104 |
| Saizonou et al., 2024 | Benin Republic | 3 | 7 | Pooled | 3.375 | P<0.01 | 1.307 |
| Tchigossou et al., 2018 | Benin Republic | 3 | 10 | Pooled | 1.585 | P≤0.05 | 0.811 |
| Tchigossou et al., 2018 | Benin Republic | 3 | 10 | Pooled | 1.1375 | P≤0.05 | 0.582 |
| Piameu et al., 2021 | Cameroon | 3 | 10 | pooled | 2.585 | P ≤ 0.05 | 1.322 |
| Piameu et al., 2021 | Cameroon | 3 | 10 | pooled | 2.7655 | P ≤ 0.05 | 1.414 |
